# Translating research findings into clinical practice: a systematic and critical review of neuroimaging-based clinical tools for brain disorders

**DOI:** 10.1038/s41398-020-0798-6

**Published:** 2020-04-20

**Authors:** C. Scarpazza, M. Ha, L. Baecker, R. Garcia-Dias, W. H. L. Pinaya, S. Vieira, A. Mechelli

**Affiliations:** 1 Department of Psychosis Studies, Institute of Psychiatry, Psychology & Neuroscience, King’s College, London, UK; 2 Department of General Psychology, University of Padova, Padova, Italy; 3 Center of Mathematics, Computing, and Cognition, Universidade Federal do ABC, São Bernardo do Campo, SP Brazil

**Keywords:** Neuroscience, Psychiatric disorders

## Abstract

A pivotal aim of psychiatric and neurological research is to promote the translation of the findings into clinical practice to improve diagnostic and prognostic assessment of individual patients. Structural neuroimaging holds much promise, with neuroanatomical measures accounting for up to 40% of the variance in clinical outcome. Building on these findings, a number of imaging-based clinical tools have been developed to make diagnostic and prognostic inferences about individual patients from their structural Magnetic Resonance Imaging scans. This systematic review describes and compares the technical characteristics of the available tools, with the aim to assess their translational potential into real-world clinical settings. The results reveal that a total of eight tools. All of these were specifically developed for neurological disorders, and as such are not suitable for application to psychiatric disorders. Furthermore, most of the tools were trained and validated in a single dataset, which can result in poor generalizability, or using a small number of individuals, which can cause overoptimistic results. In addition, all of the tools rely on two strategies to detect brain abnormalities in single individuals, one based on univariate comparison, and the other based on multivariate machine-learning algorithms. We discuss current barriers to the adoption of these tools in clinical practice and propose a checklist of pivotal characteristics that should be included in an “ideal” neuroimaging-based clinical tool for brain disorders.

## Introduction

Brain-based disorders, including psychiatric and neurological illnesses, represent 10.4% of the global burden of disease^1^, and their prevalence within the general population is thought to be increasing^2^. While the past few decades have seen significant progress in our biological understanding of these disorders, this has had little or no impact on real-world clinical practice^3–5^. This is especially the case in clinical psychiatry, where diagnostic and prognostic assessment is still based on self-reports and clinical ratings, which are associated with low inter-rater agreement and accuracy^6^. It is recognized that patients suffering from psychiatric and neurological illnesses could benefit from the translation of the research findings into clinical practice. The key question for researchers and clinicians is how to enable this^7,8^.

Over the past two decades, scientists have invested many resources in the use of brain-imaging to develop objective tests for detecting brain disorders, monitoring their progression over time and optimizing treatment. This has led to several promising findings. For example, in the field of psychiatry, structural neuroimaging has revealed widespread neuroanatomical alterations, including both transdiagnostic and disorder-specific effects^9–11^. Additionally, neuroanatomical measures have been found to account for up to 40% of the variance in clinical outcome, and can even explain some of this variance where clinical variables (e.g. diagnosis) fail to do so^12–14^. These findings have led to the suggestion that structural neuroimaging could be used to develop objective measures of psychiatric disease, in contrast with current nosological criteria which are susceptible to subjective bias^7^.

However, so far we have not been able to translate the plethora of promising findings into clinically useful imaging-based tests^5,15–17^. One of the main reasons for the current gap between research and clinical practice, is that the former has been dominated by analytical methods that only allow statistical inferences at group-level (e.g. how does the brain differ between a group of people with psychosis and a group of healthy controls?); whilst a clinician has to make diagnostic and treatment decisions at the level of the individual. In recent years, a growing number of studies have attempted to address this issue by using alternative analytical methods that allow statistical inferences at the level of the single case. A large proportion of these studies have been employing machine-learning methods to make inferences at the levels of the individual based on structural^18,19^ or functional^20,21^ neuroimaging data. This has resulted in a number of encouraging findings^22,23^. For example, machine-learning methods appear to be effective in differentiating between patients with brain illness and healthy controls, and in predicting the onset of illness and response to treatment^12,22,23^. Although this is still an emerging area of research, there is compelling evidence that neuroimaging data allow for more accurate diagnostic and prognostic inferences compared to the use of clinical and psychometric data alone^12^.

Following these encouraging findings, some research teams have been developing imaging-based tools for making inferences at the level of the individual^24–27^. Through these tools, clinicians can upload the brain images of individual patients and receive an automatic report of the brain abnormalities detected. These tools differ greatly with respect to their specific purpose (e.g. what disease is being targeted), their technical characteristics (e.g. what is the underlying statistical model), their robustness (e.g. how the tool was validated) and their availability (e.g. freely vs. commercially available). At present there is no single resource which presents all available tools and systematically compares their aims and characteristics; this means that it can be difficult for a clinician or a researcher to identify the most appropriate tool. In addition, in the absence of a systematic review of their strengths and limitations, the real translational potential of the existing tools is still unclear. To address this gap, we conducted a systematic review of available neuroimaging-based clinical tools for making inferences at single-subject level. Our first aim was to describe and compare how these tools have been developed and validated, with the ultimate goal of assessing their translational potential in real-world clinical settings. Our second aim was to use the findings to develop a checklist of the pivotal characteristics that should be included in an ideal imaging-based clinical tool for brain disorders. We hope that this review will help clinicians and researchers appreciate the aims, strengths, and limitations of the available tools and select the most appropriate option for their investigations.

## Materials and methods

### Studies selection

As the results of the current review might have health-related implications, the protocol of this review has been registered to the International Prospective Register of Systematic Reviews (PROSPERO—Registration Number: CRD42019127819). In accordance with the PRISMA guidelines^28,29^, an in-depth search was conducted on PubMed and Google Scholar databases up to February 2019. The following terms were used: (brain AND (MRI OR neuroimaging OR “magnetic resonance”) AND (“clinical tool”) AND (psychiatric OR psychiatry OR neurological OR neurology OR disease OR disorder)). All papers describing a neuroimaging-based tool developed to detect brain abnormalities in brain disorders at the level of the individual, regardless of the diagnosis, were included. Furthermore, additional relevant studies were found using different strategies. These included using the “related articles” function of the PubMed database; tracing the references from the identified papers; tracing the key references on the tool websites; and emailing the providers of the clinical tools.

### Inclusion and exclusion criteria

The following inclusion criteria were used: (i) articles presenting a neuroimaging-based clinical tool; (ii) articles presenting a tool aimed at detecting abnormalities in the brain (i.e. studies presenting a tool for detecting abnormalities in other organs, for instance the heart, were excluded); (iii) articles presenting a validation of the algorithm or technology that underlie the tool (i.e. studies applying an already validated clinical tool were excluded); (iv) articles published as original articles in peer-reviewed academic journals or conference proceedings (posters from conferences were excluded); (v) articles published or available in English.

Articles were excluded from the review according to the following a priori exclusion criteria: (i) articles that present software for analyzing neuroimaging data without a clear implementation in a translational tool (e.g. Statistical Parametric Mapping^18,19^); (ii) articles reporting studies that use non-human subjects; and (iii) studies that present clinical tools that are yet to be released.

According to our first exclusion criterion, we excluded platforms which allow the storage and analysis of individual MRI scans, using software such as Freesurfer^30^, Sienax^31^, or FSL^32^, but do not provide a clinically meaningful report including an estimate of neuroanatomical abnormalities at the level of the individual. One example is QMENTA (https://www.qmenta.com/), a cloud-based platform where different neuroimaging modalities (i.e. structural MRI, functional MRI, diffusion tensor imaging, positron emission tomography) can be stored and a different of different statistical analyses can be carried out. For instance, using QMENTA, researchers can investigate gray matter (GM) volume, cortical thickness, structural and functional connectivity, and ventricular volumetry, just to name a few of the multiple analyses which can be implemented via this platform. The advantage of using a platform such as QMENTA is the possibility to run multiple analyses simultaneously on a cloud thereby saving time. However, QMENTA does not provide researchers and clinicians with individualized reports indicating whether or not the brain under investigation deviates from those of healthy controls and what specific alternations might be driving this conclusion.

According to the same exclusion criterion, we also excluded ASSESSA PML (https://ixico.com/technology/data-platforms/assessa-platform/), a platform allowing neurologists to transfer clinical and neuroimaging data to expert neuroradiologists, who will visually inspect the scans to detect the presence of progressive multifocal leukoencephalopathy (PML), an opportunistic infection of the brain emerging as an adverse event of pharmacotherapy to treat multiple sclerosis (MS)^33^. ASSESSA PML was excluded from the current review as it is not a clinical tool that automatically extracts clinically relevant information from neuroimaging data.

### Data extraction

Two authors (C.S. and M.J.H.) extracted and checked the data independently. An additional member of the team double-checked the data in case of discordance between the first two extractions. An independent researcher oversaw the entire search procedure and randomly selected some of the articles for a random double-check. In this process, no critical issues were detected by the independent researcher. A database was created including the following characteristics: general information (authors, year of publication, name of the tool, website) and technical details regarding the tool (type of images analyzed, type of analysis performed, number of subjects used to create and validate the tool, image source, i.e. the dataset used to create and validate the algorithm, group of patients that would benefit from the tool, brain regions analyzed by the tool, validation strategy, abnormality inference strategy). Additional information regarding each tool was also recorded, including how to access it, how to use it, how the results are reported, time from images upload to report, whether the tool has been licensed, strengths and limitations.

## Results

**Fig. 1:**
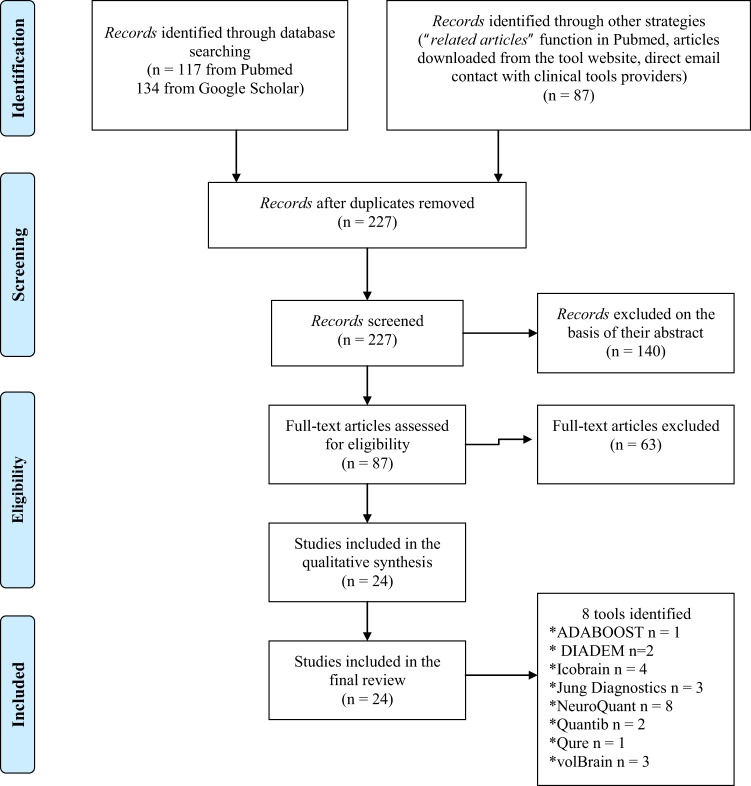
PRISMA flow chart. This figure represents the inclusion procedure used to select relevant articles following the PRISMA guidelines^28,29^.

The literature screening and final selection were performed according to the PRISMA guidelines^28,29^. This procedure is summarized in the flow diagram (Fig. 1). Applying the PRISMA procedure, a total of eight tools from 24 original articles have been included in the systematic review.

### Excluded tools

According to the PRISMA guidelines, inclusion and exclusion criteria must be decided before running the systematic search. In the current review, an additional exclusion criterion was added a posteriori: we decided to exclude tools that are no longer available. This decision was motivated by the following reasons. First, when a tool was no longer available, there was no tool-related website either; this made it impossible to collect some of the information required for the present review. Second, a tool that was no longer available was not relevant to our aim to help clinicians and researchers select the most appropriate option for their investigations. Based on this additional exclusion criterion, two tools were excluded.

The first one, ASSESSA, was initially developed to automatically provide a quantification of GM atrophy and white matter (WM) lesion volume. The focus of this tool was the quantification of hippocampal volume through the learning embeddings for atlas propagation (LEAP)^34^, an algorithm for the quantification of the regional volume which was developed to enrich clinical trials of Alzheimer’s disease in the pre-dementia phase. The clinical tool ASSESSA is no longer available.

The second tool to be excluded, called appMRI, was developed to allow for the automatic statistical analysis of hippocampal volume (http://appmri.org/en/). The tool performed an automated segmentation using FreeSurfer software and then provided a numerical output of left and right hippocampal volumes, together with normative values generated using a reference database of age-matched healthy controls. As for ASSESSA, this tool is no longer available.

### Included tools

Eight neuroimaging-based clinical tools were identified. Their technical characteristics are summarized in Table 1, while more general information, including how to use each tool and their strengths and limitations, is reported in Table 2.

**Table 1:** Technical characteristics of existing imaging-based clinical tools.

Reference	Imaging type	Type of analysis	Number of subjects used	Image source	Target disorders	Analyzed region	Validation strategy	Abnormality inference
**ADABOOST** (https://neugrid4you.eu/group/science-gateway/adaboost)								
Morra et al. (2008)^35^	3D T1	Hippocampus segmentation	200 HC for normative dataset; Training: 7 HC, 7 AD; 7 MCI Test set: leave one out approach on the training set	ADNI (http://adni.loni.usc.edu/)	AD MCI	Hippocampus	Performance compared with manually traced hippocampi	ML algorithm to compare hippocampus with the normative values
**DIADEM** (http://www.brainminer.co.uk/)								
Cardoso et al. (2012)^36^	n/a	n/a	n/a	n/a	n/a	n/a	n/a	n/a
Cardoso et al. (2015)^37^	n/a	n/a	n/a	n/a	n/a	n/a	n/a	n/a
**Icobrain** (https://icometrix.com/)								
Jain et al. (2015)^39^	3D T1+ 3D FLAIR (DICOM)	WM lesion segmentation	20 MS 10 MS	Private dataset	MS	WM	Performance compared with two software packages: LTS^90^ and lesion-TOADS^91^	Uses ML to compare WM with a priori tissue probability maps
Smeets et al. (2016)^41^	3D T1+ 3D FLAIR	Longitudinal atrophy quantification (WM and GM volume) + WM lesion segmentation	Dataset 1: 10 MS Dataset 2: 3 HC (40 scans each, longitudinal) Dataset 3: 20 MS with longitudinal scans	Dataset 1 and 3: private dataset Dataset 2: publicly available and described in^60^	MS (the atrophy quantification could also be applied to dementia)	GM and WM	Performance compared with the performance of SIENAX^31^	Not specified
Jain et al. (2016)^38^	3D T1+ 3D FLAIR	Longitudinal WM lesion segmentation	Dataset 1: 12 MS patients with longitudinal MRI (baseline, 1 year follow up) Dataset 2: 10 MS patients scanned twice (ten minutes interval) using 3 different scanners (total 60 images)	Private datasets	MS	WM	Performance compared with LTS^90^ and with experts lesions identification	Uses ML to compare WM with a priori tissue probability maps. WM lesion volume change is calculated.
Jain et al. (2019)^40^	CT	Intracranial lesion segmentation; cistern segmentation and midline shift estimation	Dataset 1: 42 subdural H; 42 epidural H; 66 intraparenchymal hemorrhages; Dataset 2: 70 cisternal compression Dataset 3: 38 patients with midline shift	CENTER-TBI study (NCT02210221)	TBI	Whole brain (specific for TBI), no tissues segmentation	Performance compared with experts’ reference segmentation	Uses ML to compare with a priori probability maps; uses ML to segment abnormalities
**Jung Diagnostics** (https://www.jung-diagnostics.de)								
Suppa et al. (2015)^27^ ARDX	3D T1	Hippocampus segmentation	44 AD 21 intermediate AD 35 non AD dementias (normative data created on 218 HC)	Private dataset	AD Dementia	Hippocampus, GM, WM, CFS	Tool performance compared with clinical diagnosis according with diagnostic criteria as gold standard	ML algorithm to compare hippocampus, GM and WM with the normative values
Suppa et al. (2015)^43^ ARDX	3D T1	Hippocampus segmentation	137 HC 103 stable MCI 95 MCI who converted to AD	ADNI (http://adni.loni.usc.edu/)	AD MCI	Hippocampus, GM, WM, CFS		
Spies et al. (2013)^42^ (Biometrica MS)	3D T1	WM lesions segmentation (T1 hypointensity)	662 HC to develop tissue probability maps Test on simulated data: 11 HC+11 MS Test: 28 HC+10 MS	662 subjects from private dataset Training: simulated data Test: private dataset	MS	WM lesions	Tool performance compared with visual rating by two independent experts	Comparison with a priori tissue probability maps
**NeuroQuant** (https://www.cortechslabs.com/products/)								
Brewer et al. (2009)^45^	3D T1 (DICOM)	Atrophy quantification; structures volume calculation and asymmetry	20 HC 20 probable AD	OASIS dataset (http://www.oasis-brains.org/)	AD	Sub-cortical structures; lateral ventricles, GM, WM, CSF	Performance compared with neuromorphometrics and with manual segmentation	Normative dataset, adjusted for age, gender and ICV. Structure volume converted in percentage of total ICV. Normative percentiles provided.
Kovacevic et al. (2009)^48^	3D T1 (DICOM)	Atrophy quantification; structures volume calculation and asymmetry	269 MCI	ADNI (http://adni.loni.usc.edu/)	MCI	Sub-cortical regions (hippocampus, amygdala, temporal horn of the lateral ventricles)	Performance compared with manual segmentation (on the 40 subjects reported in ref. ^45^)	
Azab et al. (2015)^44^	3D T1 (DICOM)	Atrophy quantification; structures volume calculation and asymmetry	46 HC 63 MTS	Private dataset	TLE	GM; sub-cortical regions, particularly hippocampus	Performance compared with the one of 12 neuroradiologists	
Farid et al. (2012)^47^	3D T1 (DICOM)	Hippocampus segmentation	116 HC 34 TLE	Private dataset	TLE	Hippocampus	Hippocampal atrophy was compared with ratings based on visual Inspection and with anatomopathological reports for 12 cases	Normative dataset, hippocampus volume converted in percentage of ICV. Normative percentiles provided
Brezova et al. (2014)^46^	3D T1 (DICOM format required)	Atrophy quantification; structures volume calculation and asymmetry	62 TBI (37 of which has longitudinal scans)	Private dataset	TBI	Sub-cortical structures; lateral ventricles, GM, WM, CSF	n/a	Normative dataset, hippocampus volume converted in percentage of ICV. Normative percentiles provided
Ochs et al. (2015)^49^	3D T1 (DICOM format required)	Atrophy quantification; structures volume calculation and asymmetry	20 HC 20 AD 20 TBI	ADNI (http://adni.loni.usc.edu/); TBI patients from a private dataset	AD; TBI	Sub-cortical structures; lateral ventricles, GM, WM, CSF	Performance compared with Freesurfer^30^	n/a
Ross et al. (2013)^51^	3D T1 (DICOM format required)	Atrophy quantification	20 HC 20 TBI	HC from ADNI (http://adni.loni.usc.edu/); TBI patients from a private dataset	TBI	Sub-cortical structures; lateral ventricles, GM, WM, CSF	Performance compared with the one of board certified radiologists to identify atrophy or ventricular enlargement by visual inspection	Normative percentiles provided. Results were consistent with parenchymal atrophy if they met one of the following criteria: (1) a parenchymal region <5th normative percentile; or (2) a ventricular region >95th normative percentile
Ross et al. (2015)^50^	3D T1 (DICOM format required)	Atrophy quantification; abnormal asymmetry; progressive atrophy	20 HC 24 TBI	HC from ADNI (http://adni.loni.usc.edu/); TBI patients from a private dataset	TBI	Sub-cortical structures; lateral ventricles, GM, WM, CSF	Performance compared with the one of board certified radiologists to identify atrophy or ventricular enlargement by visual inspection	Normative percentiles provided. Results were consistent with parenchymal atrophy if they met one of the following criteria: (1) a parenchymal region <5th normative percentile; or (2) a ventricular region >95th normative percentile
Lesion quant (no references available)	3D T1+FLAIR	WM lesions segmentation	n/a	n/a	n/a	n/a	n/a	Increased FLAIR MRI signal intensity above a set threshold when compared with surrounding tissues
PETQuant (no references available)	PET	Metabolic (FDG) and amyloid-based (Florbetapir) analysis	n/a	n/a	n/a	n/a	n/a	Visual and statistical (Z-score) comparisons of each normalized regional PET tracer value can be compared to normative population data
**Quantib** (https://www.quantib.com/)								
Vrooman et al. (2007)^53^	3D T1, Hodd-weighted HASTE; 3D T2; PD MRI (DICOM)	Tissue segmentation (GM, WM, CSF); brain lobes volumes and hippocampus segmentation	12 HC to create 59 HC to test (all females)	Rotterdam Scan Study dataset^92^	Dementia	GM, WM, CSF Lobes; hippocampus	Performance compared with manually traced brain tissues (for the 12 HC)	Atlas based *K*-nearest-neighbors for segmentation and classification; patient’s data compared with the reference centiles curve
De Boer et al. (2009)^52^	3D T1and PD-weighted; FLAIR	WM lesions segmentation	215 HC	Rotterdam Scan Study dataset^92^	MS	WM	Performance compared with manually traced WM lesions	Atlas based *K*-nearest-neighbors for segmentation and classification; WM lesions classified as GM.
**Qure** (http://qure.ai)								
Chilamkurthy et al. (2018)^25^	Non contrast CT scan	Gross abnormalities identification	291,732 CT scans to create the algorithm; 21,095 validate the algorithm; 491 to validate the algorithm	Private datasets from 20 sites in India	Intracranial hemorrhage and its subtypes; mass effect	Whole brain to detect gross abnormalities (tumors, strokes, TBI)	Comparing algorithm performance with medical reports	ML algorithm (deep learning)
**volBrain** (http://volbrain.upv.es/)								
Manjon and Coupé (2016)^26^	3D T1	Structures volume calculation and asymmetry	normative values created on 600 HC validation on 30 HC; 10 AD; 10 premature infants;	HC from IXI (http://brain-development.org/); AD from OASIS (http://www.oasis-brains.org/); Infants from BSTP (http://brain-development.org/)	AD	GM, WM, CSF^93^, TIV, cerebellum, lateral ventricles and sub-cortical structures	Performance using 50 subjects compared with Freesurfer^30^ and FSL-FIRST^32^	Normative ranges reported for structures volume (95% confidence interval).
Romero et al. (2018)^55^	3D T1+FLAIR	WM lesions segmentation	43 HC 15 MS	HC not known MS from MSSEG MICCAI Challenge 2016 (https://www.hal.inserm.fr/inserm-01397806)	MS	WM lesion, GM, WM, CSF	Performance compared with the gold standard: 7 experts consensus. Performance compared with the one of previous techniques.	Normative ranges reported for WM, GM and CSF volume (95% confidence interval). The presence of WM lesions is considered abnormal and the volume of each lesion is calculated
Romero et al. (2017)^54^	3D T1+T2	Hippocampus segmentation (both using monospectral or multispectral modality)	25 HC 5 HC	25 from the Kulaga-Yoskovitz dataset (http://www.nitrc.org/projects/mni-hisub25) 5 from the Winterburn dataset (http://cobralab.ca/atlases)	n/a	Hippocampus	Performance compared with the one of previous techniques.	Normative ranges reported for hippocampus subfields (95% confidence interval).

*AD* Alzheimer’s disease, *CT* computerized tomography, *ICV* intracranial volume, *CSF* cerebrospinal fluid, *FDG* fluorodeoxyglucose, *FLAIR* fluid attenuated inversion recovery, *GM* gray matter volume, *H* hematoma, *HC* healthy controls, *MCI* mild cognitive impairment, *ML* machine learning, *MS* multiple sclerosis, *MST* mesial temporal sclerosis, *MRI* magnetic resonance images; *PET* positron emission tomography; *T1* T1-weighted acquisition sequence, *T2* T2-weighted acquisition sequence, *TBI* traumatic brain injury; *TLE* temporal lobe epilepsy, *WM* white matter volume.

**Table 2:** Information obtained from websites.

How to access it	Report	Time to results	License	Strengths	Limitations	Notes
**ADABOOST**						
Registration to Neugrid mandatory	Report divided into three sections: (1) summary of patient’s information; (2) statistical report graphically showing the patient’s results compared with the normative range (in percentiles); (3) visual segmentation of the patient’s hippocampus in the 3 brain sections (coronal, sagittal, axial)	45 min	No license	Report easily interpreted; normative values available	ROI analysis (hippocampus only); validated on a low number of subjects; validated on neurological disorders only; inter-scanner variability not considered	n/a
**DIADEM**						
Register contacting the developers who will provide login details (log in page not present on the website); log in from the website; upload the images; download the report	Report divided into five sections: (1) summary of patient’s information; (2) image quality control summary table; (3) quality control results for both the whole brain and the hippocampus; (4) statistical report for both the whole brain and the hippocampus graphically showing the patient’s results compared with the normative range; (5) regional analysis plot graphically showing how many standard deviations below the norm each brain region volume is	More than 2 h	CE approved	Connects directly to the hospital PACS	Validated on neurological disorders only; not intuitive to use; readers should read 25 pages long manual; results not easily interpreted; inter-scanner variability not considered	DICOM conformance statement available on the website; Instruction for use (manual) available on the website
**Icometrix**						
Log in from the website; upload DICOM images; select anonymization; download the report through a link that will be sent by email	Report divided into four sections: (1) summary of patient’s information; (2) QC of the image uploaded; (3) visual results with color-coded indicators; (4) client’s demographic relevant result in normative range and percentiles (disease-specific)	1 h	FDA and CE approved	Intuitive website; report easily interpreted; normative values available; longitudinal pipeline available; automatic online images anonymization	Validated on a low number of subjects; validated on neurological disorders only; inter-scanner variability not considered	n/a
**Jung Diagnostics**						
Register contacting the developers who will provide login details; log in from the website; upload the images; download the report.	Report divided into two sections: (1) patient’s brain slices showing hippocampal atrophy; (2) statistical report graphically showing the patient’s results on a Gaussian curve where the normative ranges are indicated	n/a	CE approved	Report easily interpreted	ROI analysis; validated on neurological disorders only; inter-scanner variability not considered	A note on the website states that atrophy quantification might be important for the early diagnosis of psychiatric disorders
**NeuroQuant**						
Log in from the website, upload the images to secure server by selecting NeuroQuant report destination; download the report generated in the Picture Archiving and Communication (PAC) system	Report divided into three sections: (1) summary of patient’s information; (2) data on the brain structures analyzed, volume (cm^3^), % of ICV and 5–95% normative percentile; patient’s percentile; (3) graphical representation of the results.	8 min	FDA and CE approved; Health Canada, Australia, Korea licensed	Report easily interpreted; normative values available; longitudinal pipeline available	Cortex not analyzed; free trial not available; validated for neurological disorders only; inter-scanner variability not considered	Provides the users with recommended scanner protocol
**Quantib**						
n/a (access only via purchase. Not possible to try it, only demo available by direct call)	Report divided into three sections: (1) summary of the information provided; (2) statistical report including the absolute volume of WM, GM and CSF and the percentage of each tissue within the ICV; (3) patient’s brain slices showing GM atrophy or WM lesions.	20 min	FDA and CE approved	Reference curves differentiated between males and females; interactive report (the clinician can decide what to include or not)	Report not intuitive; validated on neurological disorders only; validated on a small number of subjects; include subjects not representative of the whole population (all females); inter-scanner variability not considered	Provides the users with recommended scanner protocol
**Qure**						
n/a (Not possible to try it, only demo available by direct call. Log in page not present on the website)	Report divided into two sections: 1) name and nature of at the abnormality detected; 2) patient’s brain slice showing the anatomical location, severity and extent of the abnormality.	n/a	CE approved	Validated on a high number of individuals (291,732 in total); dataset publicly available	CT scan only (no MRI); gross abnormalities only; inter-scanner variability not considered	Mobile notification available for neurologists when a critical abnormality is detected in a patient’s brain.
**volBrain**						
Log in from the website; upload the images (all formats- DICOM, NIfTI, zipped- accepted); download the report through a link that will be sent by email.	Report divided into three sections: (1) summary of the patient’s information; (2) statistical report including the absolute volume of each tissue and brain structure, their percentage within the ICV, their normalized volumes and an asymmetry index; (3) brain slices showing the segmented images. A graphical image representing the patient’s data compared with the normal range is available for WM lesion segmentation only.	12 min	No license	Intuitive website; normative values available; low failure rate.	Cortex not analyzed; validated on a low number of subjects; report not intuitive; not approved for medical use; validated for neurological disorders only; inter-scanner variability not considered	n/a

QC quality check, *WM* white matter, *GM* gray matter, *CSF* cerebrospinal fluid, *CT* computerized tomography, *MRI* magnetic resonance imaging, *T1* T1-weighted MRI, *FDA* Food and Drug Administration; *CE* European conformity, *ICV* intracranial volume, *ROI* regions of interest, *PACS* picture archiving and communication system, *n/a* not available.

Two of the eight tools (ADABOOST^35^ and Qure^25^) are designed to specifically perform a single type of analysis (hippocampus segmentation and gross abnormality identification, respectively). On the contrary, the other six tools (DIADEM^36,37^, Icobrain^38–41^, Jung Diagnostics^27,42,43^, NeuroQuant^24,44–51^, Quantib^52,53^, volBrain^54,55^) are designed to extract multiple types of information from the data and/or evaluate multiple disorders.

As reported in Table 2, six of the eight tools obtained at least one certification for medical use (DIADEM, Icobrain, Jung Diagnostics, NeuroQuant, Quantib, Qure). The remaining two tools are not approved for medical use. In particular, ADABOOST^35^ is present on the neuGrid platform^56^, a web portal which aims to provide automated algorithms to support the diagnostic assessment of individual patients with neurodegenerative disease from neuroimaging data. The second tool which is not approved for medical use is volBrain^26,54,55^. The website for this tool explicitly states that it was developed for research purposes, and as such does not hold any certification for medical use.

One tool (DIADEM^36,37^) has no associated references describing the underlying methodology in detail. The references that are mentioned on the website^36,37^ describe algorithms to perform parcellation and segmentation with better accuracy than previous approaches. However, it is not clear how are these algorithms are incorporated within the overall tool which performs several additional functions e.g. quantification and labeling. For this reason, we do not report the main characteristics of this tool in the following results description, as they are not present in any scientific reference.

### Target disorders

All the identified clinical tools have been developed to support the diagnosis of neurological disorders. In particular, five tools are designed to provide quantitative support to the diagnosis of dementia and in particular of Alzheimer’s disease (ADABOOST^35^, Jung Diagnostistics^27,43^, NeuroQuant^45^, Quantib^53^, volBrain^26,54^), mild cognitive impairment (MCI) (ADABOOST^35^, Jung Diagnostics^27^, NeuroQuant^48^), or other forms of dementia (Jung Diagnostics^43^). Furthermore, four tools are designed to support the diagnosis of MS (Icobrain^38,39,41^, Jung Diagnostics^42^, Quantib^52^, volBrain^55^). In addition, one tool (NeuroQuant) has a parallel version called LesionQuant which has been developed to assist the diagnosis of MS. However, no reference to a scientific publication presenting this alternative version is available on the website. Two tools supported the diagnosis of traumatic brain injury (TBI) (Icobrain^40^ and NeuroQuant^46,49–51^). Finally, one tool can be used to provide support to the diagnosis of temporal lobe epilepsy (TLE) (NeuroQuant^44,47^), and one tool (Qure^25^) is designed to identify different types of intracranial hemorrhages and mass effects in the brain.

### Type of analysis

All the identified clinical tools have been designed to perform a region of interest (ROI) analysis measuring pre-defined biomarkers for the target disorder. For instance, we know that dementia (in particular Alzheimer’s disease) is associated with atrophy of the hippocampus. Accordingly, two tools are specifically designed to focus on hippocampal volume as a biomarker of this disease (ADABOOST^35^ and Jung Diagnostics^27,43^). One additional tool is designed for the investigation of the hippocampus but has not been specifically validated in patients with dementia (volBrain^54^). Other tools support the diagnosis of dementia through the quantification of both hippocampus volume and general atrophy (NeuroQuant^45,48^, Quantib^53^, volBrain^26^). Finally, one tool performs atrophy quantification (Icobrain^41^) but has only been validated in patients with MS. As dementia might also be associated with metabolic abnormalities, one tool (PETQuant, a variation of NeuroQuant) performs automatic analysis of metabolic and amyloid based positron emission tomography (PET) images. However, no references are available for this tool.

Similarly, the main pathognomonic feature for MS is the presence of inflammatory WM lesions^57^. Accordingly, five tools are designed to perform the segmentation of WM lesions and to calculate their volume (Icobrain^38,39^, JungDiagnostic^42^, NeuroQuant—no reference available, Quantib^52^, volBrain^55^). In addition, as MS has recently been described to be associated with GM atrophy, one tool (Icobrain^41^) also provides atrophy measurements in patients with MS.

Patients with TBI present with evident traumatic lesions in the brain. A tool (Icobrain^40^) is therefore designed for intracranial lesion segmentation, cistern segmentation and the evaluation of midline shift. However, mild TBI is not associated with gross brain lesions but with subtle progressive atrophy^58^. Accordingly, a different tool (NeuroQuant^46,49–51^) has been validated to detect atrophy, structures asymmetry and/or progressive atrophy in patients with TBI.

Patients with TLE are prone to suffer from Mesial Temporal Sclerosis (MTS), involving the loss of neurons and scarring of the deepest portion of the temporal lobe, in particular, the hippocampus^59^. One tool (NeuroQuant^44,47^) is therefore designed to detect MTS in patients with TLE through the measurement of the hippocampus volume. Finally, one tool (Qure^25^) identifies gross abnormalities such as tumors and strokes.

### Brain imaging type

The vast majority of the identified tools analyze magnetic resonance images (MRI) data, in particular, T1-weighted images (ADABOOST^35^, Icobrain^38,39,41^, Jung Diagnostics^27,42,43^, NeuroQuant^44,45,47,48^, Quantib^52,53^, VolBrain^26,54,55^). However, there are a few exceptions. Four tools also require the fluid attenuated inversion recovery (FLAIR) acquisition sequence for the segmentation of WM lesions (Icobrain^38,39,41^, LesionQuant, a parallel version of NeuroQuant with no reference available, Quantib^52^, volBrain^55^). One tool (Qure^25^) analyzes non-contrast computerized tomography (CT) scans, while one tool (Icobrain^40^) requires CT scan in the case of suspected TBI. Finally, one tool (PETQuant) analyzes images acquired using positron emission tomography.

### Validation datasets and strategies

All the identified tools can be used to perform a cross-sectional analysis, and thus can be applied to support the diagnosis. Two tools (Icobrain^38,41^ and Neuroquant^46^) have also been validated on longitudinal data to predict the natural course of the disease. No tools have been validated to predict the longitudinal response to treatment.

Most tools have been validated using MRI data collected from a single dataset, either freely or private. In a small number of cases, validation is based on the use of multiple datasets. For instance, Smeets et al.^41^ (Icobrain for MS) used three datasets, two of which are private and the third one is publicly available^60^; Ochs et al.^49^, Ross et al.^50,51^ used data from healthy participants and patients with AD that were part of the ADNI dataset (http://adni.loni.usc.edu/) in combination with scans from patients with TBI which were part of a private dataset; volBrain^26,54,55^ was validated using healthy participants data from IXI (http://brain-development.org/) and from additional publicly available datasets (http://www.nitrc.org/projects/mni-hisub25; http://cobralab.ca/atlases), AD patients data from OASIS (http://www.oasis-brains.org/), infants data from BSTP (http://brain-development.org), MS data from the MSSEG 2016 (https://www.hal.inserm.fr/inserm-01397806). Qure^25^ was validated combining scans from 20 different private datasets in India. Finally, Biometrica MS^42^ (the MS version of Jung Diagnostics) combined real and simulated data. In no case, the strategy adopted to deal with the problem of different scanners and/or different acquisition parameters has been described. The strategy used to validate the tools always consisted of comparing the tool performance with the performance of the gold standard. The gold standard is mainly of three types: a ROI manual delineation by an expert; the performance of previously available software; the performance of an expert radiologist in abnormality identification by visual inspection. The tools that have been validated using the first strategy (i.e. comparison with a manual delineation of ROI) are: ADABOOST^35^, Icobrain for TBI^40^, NeuroQuant for sub-cortical segmentation^45,48^, and Quantib for both sub-cortical structure^53^ and WM lesions^52^. The tools that have been validated using the second strategy (i.e. comparison with previous software) are: Icobrain for WM lesion segmentation^38,39,41^, NeuroQuant for atrophy estimation^49^, volBrain for volumetry^26^, WM lesion segmentation^55^, and hippocampus estimation^54^. The tools that have been validated using the third strategy (i.e. comparison with visual inspection by an expert radiologist) are: Icobrain for WM lesion segmentation^38^, Jung Diagnostics for both hippocampus^27,43^ and WM lesion identification^42^; NeuroQuant for atrophy identification^44,47,50,51^. The only apparent exception is Qure^25^ where the performance of the algorithm has been compared with the results of a medical report, which in turn relies on expert visual inspection as well as other clinical data.

### Abnormality inference

All identified tools included a control group of disease-free individuals to compare the pathological brain. Five out of the eight tools (ADABOOST^35^; Icobrain^38–40^; Quantib^52,53^, Qure^25^, Jung Diagnostics^27,43^) rely on machine-learning algorithms to detect brain abnormalities as statistical deviation from the average healthy brain. Two tools rely on classical statistics to identify brains whose structures are statistically different in volume from the analogous structure in the average healthy brain: volBrain^26,54,55^ and NeuroQuant^44–51^ detect abnormalities if a brain region volume falls below the 5th percentile or above the 95th percentile of the same region in the average brain.

### Strengths and limitations

The identified tools are characterized by important strengths (see Table 2 for a tool specific description of the strengths and limitations). First, the majority of the tools rely on advanced machine-learning algorithms that offer superior ability to detect complex and distributed patterns in the data^61,62^ (ADABOOST^35^; Icobrain^38–40^; Quantib^52,53^; Qure^25^; Jung Diagnostics^27,43^). Second, most of the tools have been licensed for medical use, and this undoubtedly presents an important step toward their translational application in real-world clinical settings. Third, the time from image upload to the report receipt is less than an hour. For instance, using volBrain, results are available in 12 min; using NeuroQuant in 8 min; using Icometrix in 1 h.

However, these tools are also characterized by important limitations. First, they are validated for neurological disorders only; no tool is available for supporting the diagnosis of psychiatric disorders to date. Second, each tool performs a ROI analysis to investigate a single disorder of interest; no tool is available for investigating multiple disorders. Third, all these tools but one (Qure^25^, which relies on 291,732 images) have been validated on a small number of brain images. Although some of them used fairly large datasets to develop some normative model that could be used to detect abnormalities (e.g. *n* = 200^35^ for ADABOOST; *n* = 600 for volBrain^26^), the dataset used for validating such model tended to be much smaller (*n* = 7 MCI, *n* = 7 AD for ADABOOST^35^; *n* = 10 AD for volBrain^26^). Finally, an important limitation common to all the available tools is that none of them account for inter-scanner variability resulting from differences in scanner provider, magnetic field and acquisition parameters. This is of crucial importance to develop flexible tools that are generalizable to “unseen” scanners i.e. scanners that were not used to train the tool.

## Discussion

The current review focused on the description of neuroimaging-based analytical tools that are available to support the clinical assessment of brain-based disorders. The primary aim was to describe and compare how these tools have been developed and validated. The second aim was to use the findings to develop a checklist of the pivotal characteristics that should be included in an ideal imaging-based clinical tool. Through a systematic search of the literature, eight clinical tools were identified. The most important aspects of these tools are discussed below.

First, the available tools are targeted towards neurological disorders only. In particular, most of them were developed to assist in the diagnosis of Alzheimer’s disease and/or MS. In contrast, we could not find any tools to support the clinical assessment of psychiatric disorders. This could be explained by the current paucity of reliable imaging-based biomarkers in psychiatric disorders, where neuroanatomical alternations tend to be subtle and widespread relative to neurological disorders^63^. Second, the available tools rely on the measurement/quantification of putative biomarkers that are pathognomonic for the neurological disorder they are validated for (i.e. hippocampus volume or GM atrophy for dementia; WM hyperintensities for MS). On the one hand, this aspect is of extreme importance, as it means the tools extract the relevant information in an automated manner and provide outputs that are not affected by subjective bias. On the other hand, one could argue that the actual clinical utility of these tools is limited, because all of them have been developed to detect neurological disorders where the diagnostic accuracy is already very good. Third, all tools have been validated by comparing their performance with a gold standard, which can be of three types: (a) the performance of human experts in the manual delineation of ROI; (b) the performance of previously available software; (c) the identification of brain pathology by visual inspection. Fourth, most of the tools were trained in a single dataset, which can result in poor generalizability to unseen scanners. Related to this point, all of the tools were developed without making an explicit attempt to tackle the bias resulting from inter-scanner variability. Fifth, the tools, with few exceptions, have been created and validated using a small number of individuals, a limitation with potential implications for their reliability and generalizability. Sixth, the tools mainly rely on two strategies to detect brain abnormalities: (a) application of multivariate machine-learning algorithms to compare the patient’s brain structure with the average healthy brain (most frequent); (b) univariate comparison of the patient’s data with the average healthy brain, for instance using percentiles (5° or 95°) or confidence intervals as cut-off for detecting abnormalities.

### Adapting existing tools to psychiatric disorders: challenges

Could the existing tools be adapted to psychiatric disorders? There are many reasons why such adaptation might be challenging.

First, there are no established imaging-based biomarkers for psychiatric diagnosis^4,5^. For example, there is no single brain alteration that identifies psychosis with high sensitivity and specificity. Furthermore, the results obtained when comparing groups of psychiatric individuals against a group of healthy controls are usually unspecific. For instance, decreased GM volume of the frontal lobe has been found in schizophrenia^64^, depression^65^, PTSD^66^; this might explain the presence of cross-cutting symptoms across psychiatric disorders. Therefore, the existing tools, which analyze specific biomarkers for neurological disorders, might be difficult to adapt to psychiatric disorders.

Second, the absence of biomarkers makes the diagnosis of psychiatric disorders quite unreliable, and consequently, it can be problematic to use diagnostic labels as the gold standard to validate a tool. Thus, strategies used to validate the existing tools would be difficult to implement in the case of psychiatric disorders since: (i) there is no relevant ROIs that can be manually traced; (ii) there are no software that reliably identifies psychiatric individuals at the level of the single subjects; (iii) psychiatric pathology cannot be identified by brain visual inspection. To create a tool that can be reliably applied to psychiatric research, an alternative validation strategy and gold standard would need to be identified.

Third, we need to consider the issue of disease heterogeneity. Although both psychiatric and neurological disorders tend to be heterogeneous in terms of clinical presentation, naturalistic course of the illness and treatment response^67–69^, neurological disorders are characterized by more specific and reliable neural correlates than psychiatric disorders. For example, atrophy of the hippocampus in Alzheimer’s disorder is evident above and beyond the neuroanatomical heterogeneity of the disease. The same cannot be said for the neuroanatomical alterations that are typically observed in psychiatric disorders. Here, neuroanatomical alterations tend to be subtle and widespread, making the discrimination between normal heterogeneity and pathological heterogeneity more challenging^63,70,71^. This means that the adaptation of existing tools to psychiatric disorders would require careful consideration of the issue of heterogeneity^72^.

Finally, we need to pay attention to how statistical inferences about the presence/absence of neuroanatomical abnormalities are made. As the neural correlates of psychiatric disorders are subtle, diffuse and complex, abnormality inferences that rely on classical statistics (e.g. percentiles) are likely to be highly prone to false negative findings. When adapting the existing tools to psychiatric disorders, therefore, it would be appropriate to adopt statistical models that can detect high orders of complexity and abstraction in the data. In this scenario, the application of advanced machine-learning methods, such as convolutional neural networks, is a promising strategy^73,74^.

In short, if the scientific and clinical psychiatric community is still devoid of a neuroimaging-based clinical tool to enrich the diagnostic pathway, the main reason appears to be the complexity of the problem at hand. Compared to neurological disease, psychiatric disorders are characterized by higher levels of etiological, phenotypic and neurobiological overlap, and heterogeneity^75^; this makes the task of developing reliable imaging-based biomarkers a significantly greater challenge.

### What would an ideal clinical tool for brain disorders look like?

**Fig. 2:**
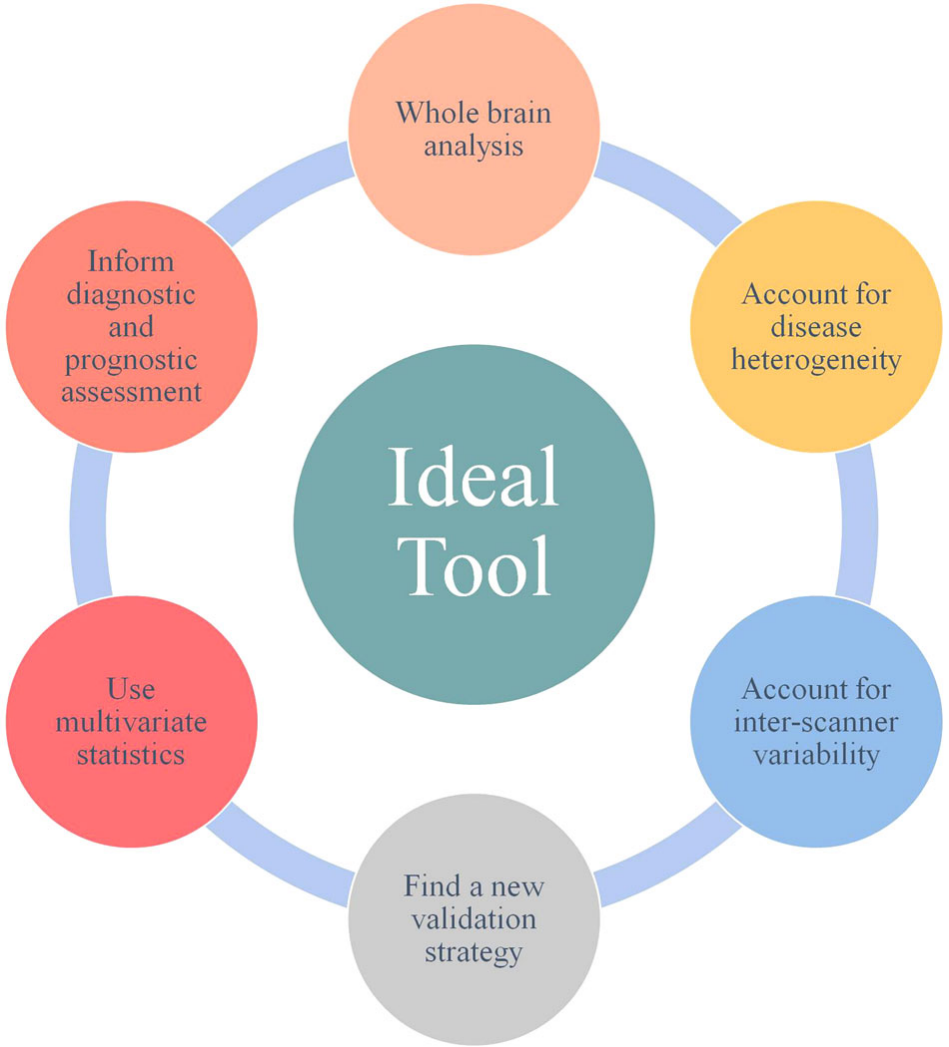
Proposal for an ideal imaging-based clinical tool. This figure summarizes the characteristics of an ideal clinical tool to assist the clinical assessment of psychiatric disorders.

In this last section, we propose several pivotal characteristics that should be included in an ideal imaging-based clinical tool (graphically represented in Fig. 2) to assist the clinical assessment of psychiatric disorders.

- *From a region-of-interest to whole-brain approach*: Existing tools for neurological disorders use a region-of-interest approach to detect localized alternations. Considering the subtle and widespread neural correlates of the psychiatric disorders^22,71,76^, the ideal clinical tool should not restrict its analysis to a single or few regions; instead, it should analyze the whole-brain to exploit all the available neuroanatomical information.
- *Accounting for disease heterogeneity*: As etiological, neurobiological and phenotypic heterogeneity is a key aspect of brain disorders^67,70,77^, the ideal tool should be created and validated on a sample which is large enough to capture such variability. While the required number of subjects depends on the heterogeneity of the disease under investigation, this is likely to be in the order of hundreds or even thousands for most brain disorders. In addition the sample size should be large enough to allow the investigation of gender-specific and age-specific effects within a clinical population of interest. As the number of subjects used to create and validate the tool increases, so does the sample heterogeneity due to the loosening of inclusion criteria. On the one hand, higher levels of heterogeneity make the creation of an accurate tool more challenging, as the model needs to be able to distinguish between normal heterogeneity and pathological heterogeneity^78,79^. On the other hand, larger samples are more likely to have a normal distribution and be representative of the clinical population of interest, and as such carry greater translational potential in real-world clinical practice.
- *Accounting for inter-scanner variability*: As the ideal clinical tool is supposed to handle MRI scans of individuals from different clinicians/hospitals/countries, it should be able to estimate and account for differences in scanner provider, magnetic strength field and acquisition parameters. This is especially important for psychiatric disorders, where the effects of interest are subtle and, therefore, inter-scanner variability can be much greater than disease-related variability^71,80,81^.
- *The importance of validation*: Since the validation strategies used for neurological disorders—where we have a few established diagnostic biomarkers—cannot be applied to psychiatric disorders, it is of pivotal importance to identify an alternative strategy to validate the tool. A possible solution might be to switch the focus from diagnostic to prognostic assessment and establish a prospective link between neuroanatomical alterations and clinical outcomes^12^. As an example, studies have shown that neuroanatomical alternations in patients at high clinical risk of developing psychosis are predictive of future transition to the illness^82^; as a further example, cortical folding defects in people with a first episode of psychosis have been found to be predictive of future response to pharmacological treatment^83^. The use of clinical outcome measures could, therefore, provide an alternative validation strategy for tools targeting psychiatric disorders.
- *Using advanced multivariate statistics to capture abstract and complex patterns in the data*: As the neural correlates of psychiatric disorders are subtle and distributed, the ideal clinical tool should use multivariate rather than univariate algorithms. In addition, in light of current conceptualizations of psychiatric and neurological illnesses as network-level disorders of the brain^84,85^, the ideal clinical tool should be able to capture multivariate interactions with high levels of abstraction and complexity. There are several statistical and machine-learning methods which could be used to achieve this. For example, deep learning is a family of algorithms that can detect high orders of complexity and abstraction in the data and make inferences at the level of the individual with greater precision than ever before^62^. In light of these qualities, deep learning algorithms are attracting significant interest in neuroscience including psychiatric and neurological research^86^.
- *Informing diagnostic and prognostic assessment*: The ideal tool would assist clinicians through the complex tasks of clinical assessment and prognostic decision-making. Thus, the tool should indicate the likelihood of a certain diagnosis or a certain clinical outcome. This could be achieved by matching the neuroanatomical abnormalities identified in a patient with the neuroanatomical alterations that are known to be associated with a certain psychiatric disorder (in the case of diagnostic inference) or a certain clinical outcome (in the case of prognostic inference). A high/low match score would indicate that an individual presents with neuroanatomical changes that are typical/atypical of a certain psychiatric or neurological disorder a certain clinical outcome.

## Conclusions

A pivotal aim of neuroimaging research is the development of clinical tools that can support clinical decision-making by producing accurate, objective, and real-time outputs from neuroimaging data^17^. The results of this review indicate that there is a very limited number of clinical tools available to support the diagnosis of neurological disorders, while there are none for psychiatric disorders. In addition, only two of the available tools have been validated using longitudinal datasets, and are therefore suitable for prognostic assessment. The majority of the available tools (4 out of 7) make use of multivariate machine-learning methods, which allow inferences at the level of the individual and as such open up new possibilities in personalized medicine^87^. However, the results of such methods should be interpreted with caution^22^ as they can be over-optimistic due to a combination of small sample sizes and less-than-rigorous methodologies^78^. A further complication is that several genetic and environmental factors that can affect the structure of the human brain without necessarily leading to pathology^88^. This means one must avoid the pitfall of considering structural brain abnormalities pathological per se should be avoided; instead, researchers and clinicians must interpret the output of a machine-learning model in light of the patient’s clinical history and symptomatology^89^. A related consideration is that the ideal tool should not be limited to the examination of brain abnormalities, but might also benefit from the integration of potentially valuable information such as duration of illness and symptomatic presentation^90^.

In conclusion, we envisage a future in which imaging-based tests will complement traditional clinical assessments of psychiatric and neurological disorders, leading to biologically informed diagnosis, monitoring and treatment of individual patients. Before this vision can be realized, however, several outstanding challenges need to be addressed; these include, for example, the issues of neuroanatomical heterogeneity, inter-scanner variability, and validation. We hope the observations and suggestions included in the present article will help researchers realize this vision in the future.
